# Differential Talin cleavage in transformed and non-transformed cells and its consequences

**DOI:** 10.3389/fcell.2024.1430728

**Published:** 2024-07-17

**Authors:** Xian Hu, Salma Jalal, Mingxi Yao, Oddmund Bakke, Felix Margadant, Michael Sheetz

**Affiliations:** ^1^ Center for Cancer Cell Reprogramming, Faculty of Medicine, University of Oslo, Oslo, Norway; ^2^ Department of Biosciences, University of Oslo, Oslo, Norway; ^3^ Mechanobiology Institute, National University of Singapore, Singapore, Singapore; ^4^ Department of Biomedical Engineering, Southern University of Science and Technology, Shenzhen, Guangdong, China; ^5^ Molecular Mechanomedicine Program, Biochemistry and Molecular Biology Department, University of Texas Medical Branch, Galveston, TX, United States

**Keywords:** mechanobiology, TIRF and FRAP, talin cleavage, focal adhesion, cancer biology, calpain

## Abstract

This study investigates differences in focal adhesion (FA) morphology and Talin cleavage levels between transformed and non-transformed cell lines. Utilizing fluorescently tagged wild-type Talin and Talin mutants with calpain cleavage site mutations, FA structures were visualized. Mutations in different Talin cleavage sites showed varying impacts on FA morphology and distribution across melanoma cell lines (Meljuso, A375P, A2058) and a non-transformed cell line (HFF). Western blot analysis, ratiometric fluorescence intensity-based measurements, and FRAP experiments revealed higher Talin cleavage levels within FAs of transformed cell lines compared to non-transformed cells. Additionally, growth assays indicated that reducing calpain cleavage levels attenuated transformed cell growth. These findings suggest that Talin cleavage level is crucial for FA morphology and assembly, with higher levels observed in transformed cells, influencing their growth dynamics.

## 1 Introduction

Cancer cells are known to be able to grow on soft surfaces, but non-cancer cells cannot proliferate well on soft surfaces (Frisch and Francis, 1994). This characteristic has long served as a hallmark of carcinogenesis and forms the basis of the widely used soft agar colony formation assay for cancer diagnosis. This raised intriguing questions about the mechanosensing capabilities of cancer cells. Recent investigations have revealed that many cancer cell lines lack crucial components of the contractile unit machinery (EGFR, HER2, ROR2, AXL, Myosin IIA, Tpm2.1, Tpm3, FLNA and α-actinin 1 and 4) essential for cellular mechanosensing (Wolfenson et al., 2016; Yang et al., 2020). Remarkably, upon restoration of these missing contractile components, cancer cells lose their ability to proliferate on soft surfaces, indicating a restored mechanosensing capacity. These restored cancer cells start to have increased levels of apoptosis on soft surfaces, a phenomenon commonly referred to as anoikis: the substrate rigidity triggered apoptosis (Frisch and Screaton, 2001). Anoikis is particularly important in cancer research as it plays a role in preventing the spread of cancer cells to other tissues.

One of the pivotal enzymes implicated in triggering anoikis is Death-associated protein kinase 1 (DAPK1) (Kwon et al., 2016). Inhibition of DAPK1 activity has been shown to enable non-transformed cells to grow on soft surfaces, highlighting its crucial role in anoikis. DAPK1 has been linked to various cytoskeletal proteins and cellular motility processes, including its ability to phosphorylate tropomyosin1 (Tpm1.1) and myosin light chain. Notably, the recruitment of DAPK1 into FA has been found to be dependent on Talin cleavage by the calcium-dependent protease calpain. The deficiency of Talin1 in cells has been associated with protection from apoptosis on soft surfaces, underscoring the importance of calpain-mediated Talin cleavage in regulating cellular responses to substrate rigidity (Qin et al., 2022).

Calpain is a calcium dependent protease that cleaves FA proteins in favor of FA disassembly.The turnover of FA are pivotal events that activate signaling pathways regulating fundamental cellular processes such as migration and proliferation in both normal and transformed cells.Talin is a large (−270 kDa; −2540 amino acids) mechanosensitive protein that serves as a molecular scaffold in FA, connecting integrin cell surface receptors directly to the intracellular cytoskeleton and recruiting many other FA proteins (Yan et al., 2015; Hu et al., 2016; Yao et al., 2016; Hu et al., 2017). Talin is one of the main targets of calpain cleavage in FAs and can be cleaved at two different sites: the N-terminal cleavage site L432 cutting Talin into the head domain and the rod domain, and the C-terminal cleavage site: E2492 (Bate et al., 2012). Mutation on the Talin cleavage site impairs the cell spreading dynamics and migration. Non-cleavable Talin reduces the cell spreading area, and cells with non-cleavable Talin have reduced protrusion-retraction cycles at the edge of lamellipodia upon EGF induced motility activation (Bate et al., 2012; Saxena et al., 2017; Strömblad, 2022). Additionally, the cleavage of Talin plays a more crucial role for cells spreading when cells are plated on median to soft surface but less when cells are plated on rigid surface (Saxena et al., 2017).

There is a complex relationship between the cleavage of Talin and adhesion strength/tension. In the case of soft surfaces, the turnover of the adhesions is more rapid and there are fewer stress fibers. On rigid surfaces, the turnover is slower with more stress fibers. Nevertheless, the total force on the adhesions is the same on soft and rigid surfaces (Doss et al., 2020). In the earlier studies of Saxena et al., 2017 the non-cleavable Talin causes a slow recruitment of Talin and force on pillars as cells spread. Despite these insights, the precise implications of Talin cleavage on cancer cell behavior remain elusive.

In this study, we delve deeper into understanding the impact of calpain-mediated Talin cleavage on transformed cell behavior, with a focus on its role in the FA dynamics and cell growth rate. Transformed cells exhibit altered Talin expression levels and distinct FA morphology compared to non-transformed cells, suggesting a pivotal role of Talin cleavage in regulating FA dynamics in cancer (Te Boekhorst et al., 2022; Sharbatoghli et al., 2023). Through examination using three distinct methods, it becomes evident that transformed cells exhibit elevated Talin cleavage activity compared to non-transformed cells. Importantly, inhibition of Talin cleavage has been shown to attenuate the proliferation rate of cancer cells, highlighting the potential therapeutic implications of targeting Talin cleavage in cancer treatment strategies.

## 2 Methods

### 2.1 Cell culture, drug and plasmid

Cells were maintained in a humidified incubator set at 37°C and atmospheric 5% CO_2_ and cultured in Dulbecco’s Modified Eagle’s Medium (DMEM, Invitrogen) high glucose supplemented with 10% Heat Inactivated Fetal Bovine Serum (HI-FBS, Invitrogen) and 1 mM sodium pyruvate (Invitrogen). ALLN inhibitor treatment was previously described Lynch et al. (Lynch et al., 2013).

Dr. Paramasivam Kathirvel from the MBI cloning core facility generated: EGFP-Talin Rod-mCherry from the deletion of head domain (AA 1–434) from EGFP-N-Talin1-C-mCherry (Margadant et al., 2011). Plasmids encoding full length Talin tagged with GFP and mCherry with mutation(s) at either or both calpain binding sites at the N- and C- terminals were kind gifts from the Goult Group (School of Biosciences, University of Kent).

### 2.2 Introduction of siRNA and plasmid into cells

For genetic silencing of Talin-1, cells were seeded in 35 mm dishes on Day 0. On Days 1 and 2, melanoma cell lines were transfected with 20 μM of TLN1 siRNA (Dharmacon, ON-TARGETplus Non-targeting pool siRNA, catalog no. L-012949-00-0010) using Lipofectamine RNAiMAX (Invitrogen) following the manufacturer’s instructions. HFF were transfected with 3 doses of 20 μM of TLN1 siRNA over 3 days.

Transient transfection of DNA plasmids into HFF was done by electroporation (Neon transfection system, Life Technologies) following manufacturer’s instructions with electroporation conditions of two pulses of 1150 V for 30 ms.

### 2.3 Immunoblotting

Cells were lysed with RIPA buffer (Sigma) and extracted proteins were separated by 4%–20% gradient SDS-polyacrylamide gel electrophoresis (Thermo Fisher Scientific) and transferred to a 0.45 μm PVDF membrane (Bio-Rad) at 100 V for 1.5–2 h. Membranes were blocked with 5% non-fat milk (Bio-Rad) in TBST for 1 h at Room Temperature (RT) before incubation with the appropriate primary antibody: mouse anti-GAPDH (6C5, Santa Cruz Biotechnology/Abcam) at a dilution of 1:3,000, mouse anti-α-tubulin (DM1A, Sigma) at a dilution of 1:10,000, mouse anti-Talin-1 (97H6, Bio-Rad) at a dilution of 1:1,000 in 5% non-fat milk in TBST at 4°C overnight. After washing in TBST, the membrane was incubated with an anti-mouse HRP-conjugated secondary antibody (Bio-Rad) at a dilution of 1:2000 in 2.5% non-fat milk in TBST. The membrane was then processed for ECL detection (Bio-Rad) and chemiluminescence was detected in the Bio-Rad ChemiDoc Imaging System.

### 2.4 Optical microscope and image processing

Live Cell Imaging Experiment: Cells were transfected and seeded on glass bottom images (IBIDI Cat.No: 81218-200) with various plasmids 24 h before imaging at a confluency of around 40%. FA intensity ratio image and FRAP data imaged by Gataca Systems iLas2 Ring TIRF system with FRAP module. The system is based on a Olympus IX81 body and equipped with dual cameras with W-VIEW GEMINI beam splitter from Hamamatsu and two Prime95 sCMOS from Teledyne Photometrics. The TIRF FRAP images were captured using a 60X/1.49 oil-immersion objective, while the growth assay cell counting experiments were captured using a ×20/0.40 objective.

Image Analysis: Image analysis in this project was conducted by ImageJ macro script. For the FRAP data analysis, three ROI (region of interest) were selected manually by the user: 1) ROI(bleach): the ROI of the area that is being bleached. 2) ROI (background): the ROI of an empty area in the same image. 3) ROI (bleach): the ROI of an area on the cell that is not being bleached in the same image. The FRAP curve is measured and plotted by the following equation: (Intensity of ROI(bleach)—Intensity of ROI (background)) divided by (Intensity of ROI (background)-Intensity of ROI (background). For the FA ratio calculation, the images were preprocessed by background subtraction and were segmented with Otsu Threshold (Fiji built-in function).

## 3 Results

### 3.1 Different cell morphology and FA morphology in transformed vs. non transformed cells

**FIGURE 1:**
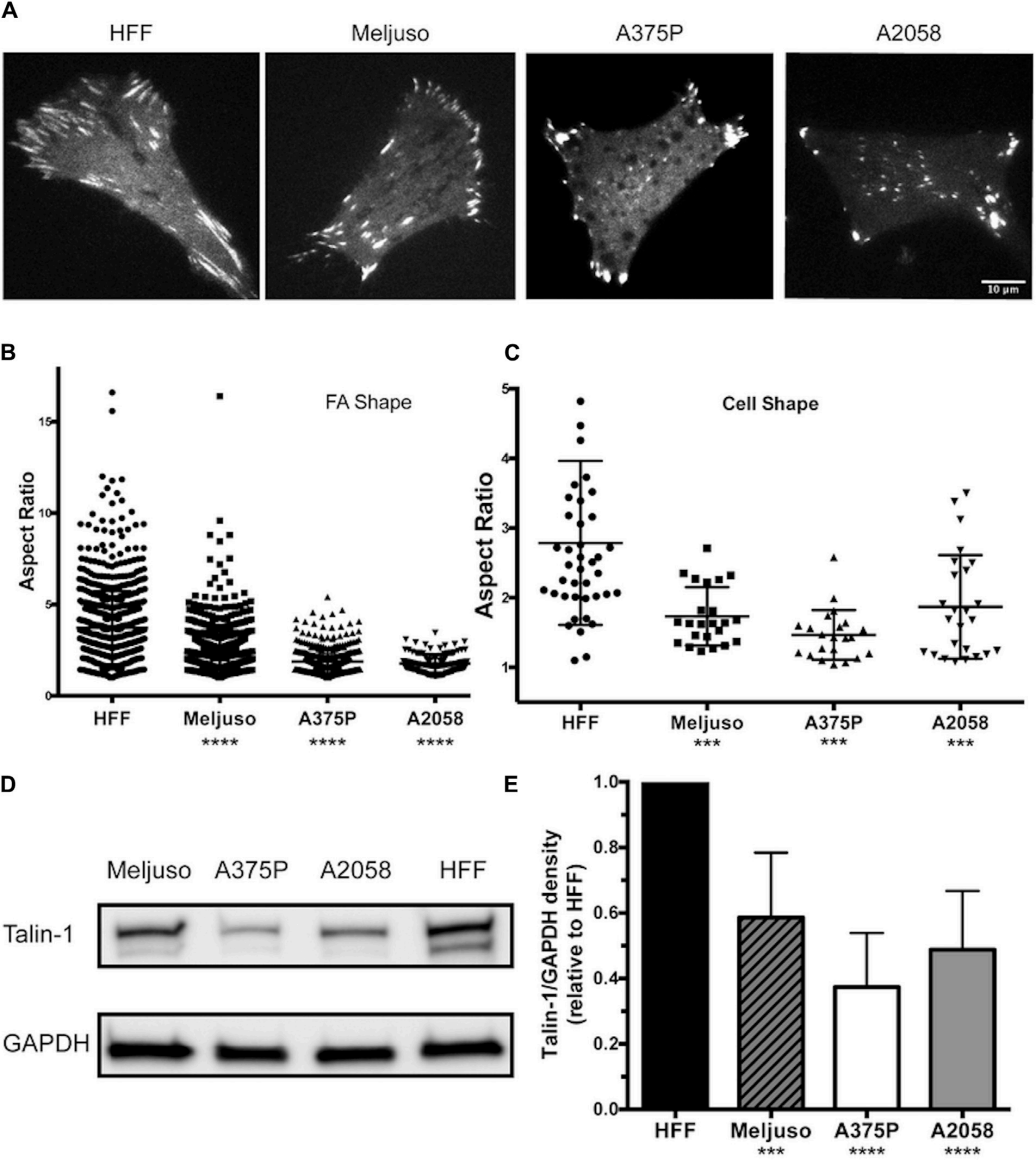
Melanoma cells exhibit Reduced FA Size and Lower Endogenous Talin-1 Levels Compared to Non-transformed Fibroblasts.**(A)** Representative images illustrating the distribution of focal adhesions in cells expressing fluorescently tagged full-length wild-type Talin-1 (GTC). Scale bar, 10 μm. **(B)** The aspect ratio of focal adhesions indicates that HFF cells possess longer focal adhesions compared to transformed cell lines (Meljuso, A375P, and A2058 cells). **(C)** The aspect ratio of cells highlights increased polarization in HFF cells compared to transformed cell lines (Meljuso, A375P, and A2058 cells). **(D)** Representative western blot images depicting Talin-1 and GAPDH expression in total protein lysates extracted from HFF, A375P, Meljuso, and A2058 cells. **(E)** Bar chart presenting the ratio of Talin-1/GAPDH density expressed in melanoma cell lines normalized by Talin-1/GAPDH expression in HFF from the same blot. The graph is plotted with mean and standard deviation from at least 6 sets of blots. Dunnett’s multiple comparison test was employed to assess significance between Talin-1 expression in melanoma cell lines compared to the HFF control: *P<0.05, **P<0.01, ***P<0.001, ****P<0.0001.

Previous studies have provided both direct and indirect indications that FA structure and dynamics differ between transformed and non-transformed cell lines. In our investigation, we employed fluorescently tagged Talin-1 to visualize FA structures, facilitating the subsequent assessment of FA and cell morphology variations among three melanoma lines (Meljuso, A375P, and A2058) and a non-transformed cell line, human foreskin fibroblast (HFF) (Figure 1A). Notably, the average number of FAs per cell did not exhibit a significant difference across the 4 cell lines (Data not shown). However, distinct differences were observed in the aspect ratio of FAs and cell morphology, with HFF demonstrating significantly higher values compared to the three melanoma cell lines (Figures 1B,C).

Of particular interest, total protein lysates extracted from HFF cells revealed significantly higher levels of endogenous Talin-1 compared to those obtained from melanoma cells (Figures 1D,E). These findings shed light on potential correlations between Talin-1 expression, FA characteristics, and cell morphology, highlighting intriguing distinctions between normal and melanoma cell lines.

These data collectively illustrate that melanoma cells display smaller FAs and lower endogenous Talin-1 levels in comparison to non-transformed fibroblasts, suggesting potential implications for cell adhesion and signaling dynamics in these distinct cellular states.

### 3.2 Talin and its cleavage affect the FA morphology and distribution in cells

**FIGURE 2:**
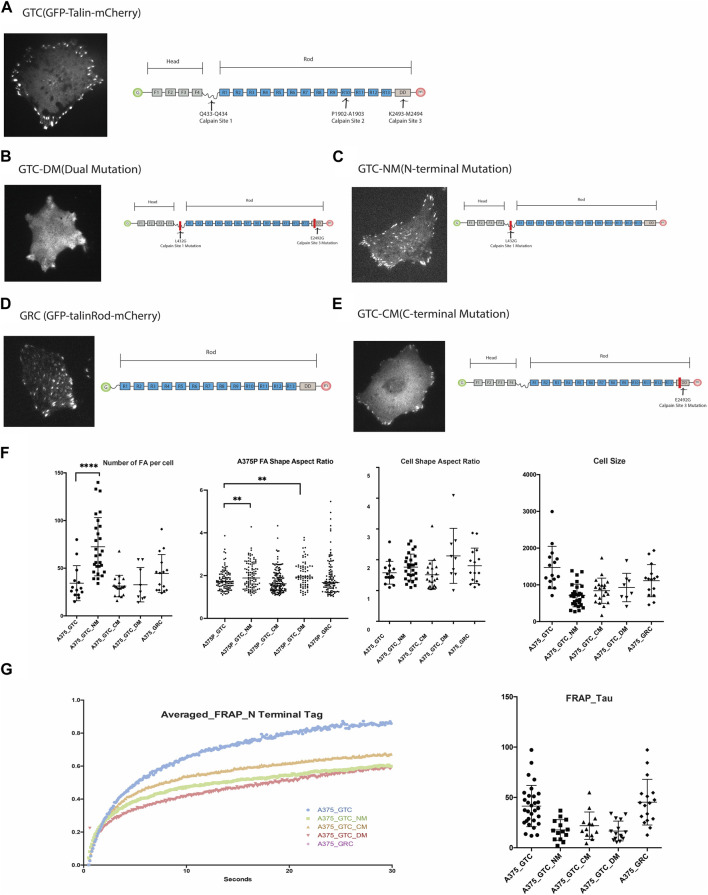
**(A)**. Expression of GTC (GFP-Talin(wildtype)-mCherry) in 375P cells and its construct design. **(B)**. Expression of GTC-DM (GFP-Talin Dual Mutation, L432G and E2492G) in A375P cells and its construct design illustration. **(C)**. Expression of GTC-NM (GFP-Talin N-Terminal Mutation, L432G) in A375P cells and its construct design illustration. **(D)**. Expression of GRC (GFP-Talin Rod Domain-mCherry) in A375P cells and its construct design illustration. **(E)**. Expression of GTC-CM (GFP-Talin C-Terminal Mutation, E2492G) in A375P cells and its construct design illustration. **(F)**. Quantification and comparison of FA number, aspect ratio, cell shape, and cell size when different mutants are expressed in A375P cells. **(G)**. FRAP data of N terminal Tag of GTC, GTC-NM, GTC-CM, GTC-DM and GRC expressed in A375P cells.

Although three calpain binding and cleavage sites have been identified along Talin at Q433-Q434, P1902-A1903, and K2493-M2494 [4], loss-of-function mutations have only been produced and validated for the N-terminal (Q433-Q434) and C-terminal (K2493-M2494) sites thus far. We obtained full-length Talin and Talin mutants tagged with GFP and mCherry at the N- and C-terminals, respectively, from the Goult Group at the School of Biosciences, University of Kent. Additionally, we generated a truncated construct lacking the head domain of Talin, GFP-Talin Rod-mCherry (Figure 2), to examine the morphology of FAs associated with these constructs. The Talin Head domain is dispersedly located in the cytosol and is considered not to contribute to the function of FA; therefore, it was not included in this study (Ref: Siming Paper).

List of constructs used in this study:1.GTC (GFP-Talin (wildtype)-mCherry). 2.GTC-DM (GFP-Talin Dual Mutation, L432G and E2492G).3.GTC-NM (GFP-Talin N-Terminal Mutation, L432G).4.GRC (GFP-Talin Rod Domain-mCherry).5.GTC-CM (GFP-Talin C-Terminal Mutation, E2492G).

To initiate the investigation into the impact of each mutant on FA and cell morphology, we first quantify and compare the morphological parameters of FAs and cells when different mutants are expressed (Figure 2). When both calpain sites were mutated (GTC-DM), the cells became smaller, adopting a more dendritic-like shape, and the boundary between FAs and cytosol became unclear, making it challenging to accurately define the FA area. Similarly, when the N-terminal Talin cleavage site was mutated (GTC-NM), the cells also became smaller. The expression of this mutant significantly increased the number of FAs in the cells, particularly in the center. These FAs, which would normally dissipate as the cell leading edge moves away from them, persisted. This observation is consistent with the role of the N-terminal cleavage site in generating truncated Talin Rod and Head domains, which are known to be crucial for FA turnover.

Overexpression of Talin Rod in the cell (GRC) also resulted in an increased number of FAs located in the center of the cells, which were positive for GRC. In cells transfected with GTC-NM and GRC, we observed that focal adhesions at the cell periphery exhibited a larger aspect ratio (AR) compared to those located centrally. The difference in AR between peripheral and central focal adhesions was more pronounced in cells expressing GTC-NM (bearing an N-terminal mutation, L432G), than in those expressing GRC (a construct pertaining to the Talin rod domain). A higher aspect ratio is indicative of more elongated focal adhesions, which typically suggests exposure to greater mechanical force (Supplementary Figure S1).

The FA aspect ratio is significantly larger in A375P cells transfected with the Talin N-terminal mutation (NM) and Talin dual mutation (DM). Cells transfected with the Talin rod domain exhibit a greater number of FAs with larger aspect ratios, resulting in a shift in the population distribution shape as indicated in Figure 2, panel F. These results align with expectations: reduced Talin cleavage is projected to result in a lower FA disassembly rate, thereby producing longer FAs (with a larger aspect ratio). Transfection with the Talin C-terminal mutation (CM) does not significantly affect the FA aspect ratio, but the edge of the FA appear less defined as compare to other mutants. This is in line with the previous report that the N-terminal cleavage site is the most active, while the C-terminal site exhibits less activity. (Figure 2F).

FAs are highly dynamic organelles, and if the mutants alter the morphology and distribution of FAs and cells, they should also affect the dynamics of FAs. To test this hypothesis, we conducted a TIRF-FRAP experiment of the FAs when these five constructs are expressed in A375P cells (N-terminal tagged GFP). As anticipated, the different Talin cleavage mutants exhibit distinct FRAP profiles compared to wild-type Talin. The N-terminal tag of the Talin Rod domain (GRC) displays a similar FRAP recovery time (Tau) compared to the wild-type Talin molecule, whereas all the other three cleavage site mutations demonstrate a smaller recovery time (Tau) compared to the wild-type.

FRAP assays typically quantify the rate of fluorescence recovery after photobleaching, which is indicative of the diffusion rate. This replenishment rate of the bleached protein is characterized by the shape of the recovery curve, often quantified by the FRAP recovery time constant, Tau. A smaller Tau value suggests a quicker recovery speed post-bleaching, reflecting a faster rate of diffusion. Rapid diffusion may be attributed to features such as a smaller molecular weight or a more hydrodynamic protein structure. In the case of the Talin cleavage mutant, it is improbable that a reduction in molecular weight is responsible for any observed increase in diffusion rate since the mutation decreases the likelihood of Talin cleavage. Instead, it is conceivable that the cleavage site mutation prompts a conformational alteration in the molecule that enhances its hydrodynamic properties.

### 3.3 Transformed cell and non-transformed cells have different Talin cleavage level

Previous studies have implicated Talin cleavage in the regulation of cell anoikis and FA turnover (Saxena et al., 2017). To examine this further, we assessed Talin cleavage levels in one non-transformed cell line, HFF, and three transformed cell lines (Meljuso, A375P, A2058).

**FIGURE 3:**
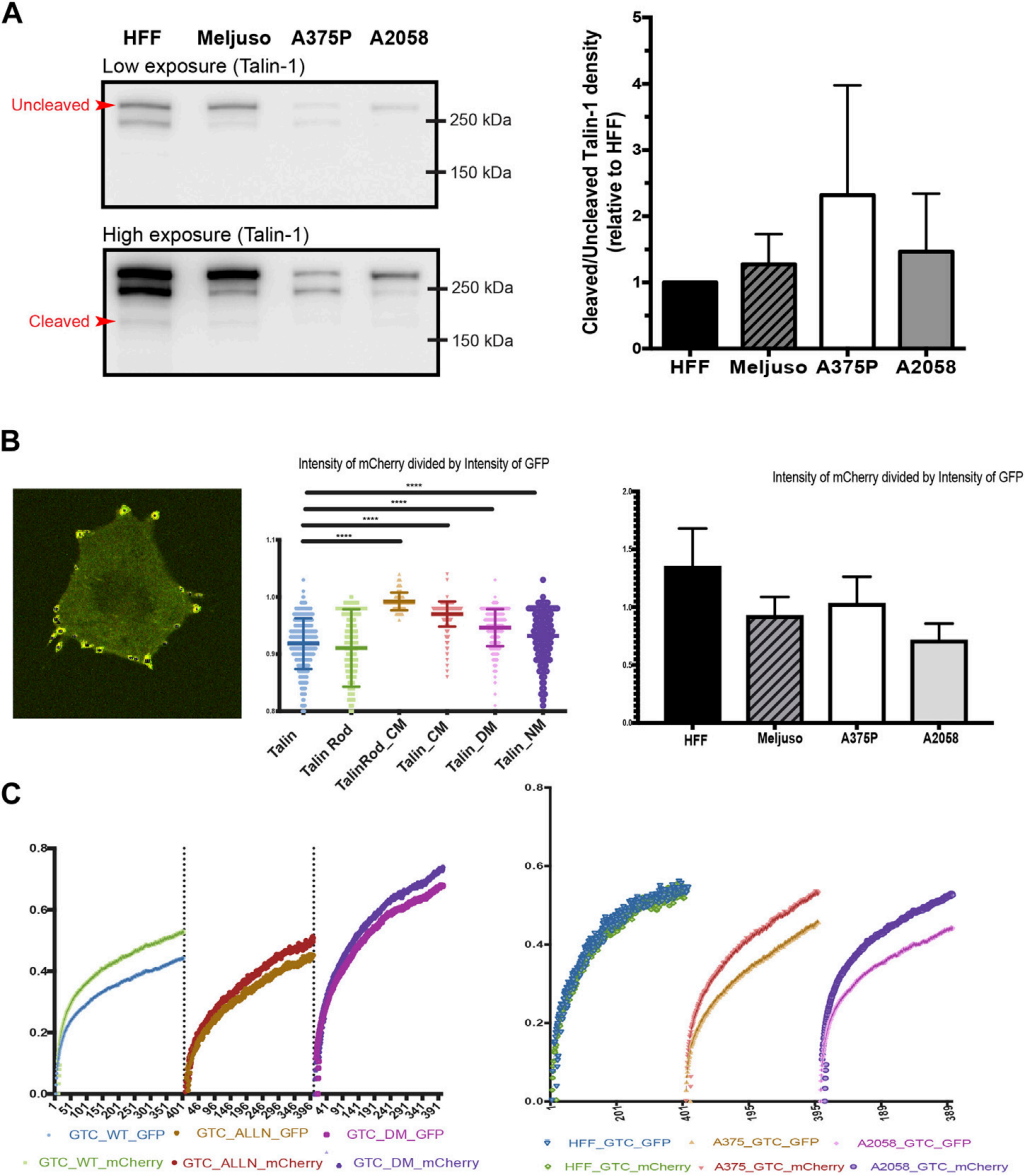
**(A)**. Left: Representative western blot of Talin expression in total protein lysates taken from HFF, A375P, Meljuso, and A2058 cells. The higher exposure image shows visible bands of cleaved Talin. Right: Bar chart showing the ratio of uncleaved/cleaved Talin expressed in melanoma cells normalized by the ratio in HFF from the same blot, plotted with mean and standard deviation from 5 sets of blots. Dunnett’s multiple comparison test was used to assess significance between uncleaved/cleaved Talin expression in melanoma cell lines compared to HFF as a control. **(B)**. Left: Representative image of cells with fluorescently transfected GFP-X (name of mutant)-mCherry construct. Middle: Ratio of mCherry intensity divided by GFP intensity in individual FAs with different mutants. Right: Ratio of mCherry intensity divided by GFP intensity in individual FAs with different cell lines. **(C)**. Left: Average FRAP curve of GFP and mCherry in individual FAs with different mutants. Right: Average FRAP curve of GFP and mCherry in individual FAs with cell lines.

Western blot analysis was initially employed to evaluate the overall expression levels of Talin (Kerstein et al., 2017). Our results revealed slightly elevated levels of Talin cleavage in all three transformed cell lines compared to HFF cells (Figure 3A). Given that Western blot analysis provides information on total protein expression but does not specifically assess Talin cleavage within specific FAs, we adopted a ratiometric fluorescence intensity measurement approach. This method involved measuring the ratio of fluorescence intensity between the N-terminal GFP tag and the C-terminal mCherry tag of Talin within FAs. A ratio close to one indicates minimal Talin cleavage.

To validate this method, we transfected A2058 cells with different Talin cleavage mutants (Figure 3B, middle panel). The results demonstrated that mutations disrupting calpain cleavage sites led to significantly higher ratios of mCherry to GFP fluorescence intensity compared to wild-type Talin. (The average ratio of mCherry divided by GFP in A2058 with wild type Talin is 0.918, that of A2058 with Talin Rod CM(C-terminus Calpain site) mutant is 0.992, of A2058 with Talin CM(C-terminus Calpain site) mutant is 0.970, of A2058 with Talin DM(dual Calpain site) mutant is 0.948 and of Talin NM(N-terminus Calpain site) mutant is 0.931.).

Subsequently, we applied this method to HFF cells and the three transformed cell lines (Meljuso, A375P, A2058). Consistently, all three transformed cell lines exhibited statistically lower ratios compared to HFF cells (Figure 3B, Right), indicating higher Talin cleavage levels within individual FAs.

To corroborate these findings and address potential limitations of fluorescence intensity-based measurements, we performed Fluorescence Recovery After Photobleaching (FRAP) experiments using Total Internal Reflection Fluorescence (TIRF) microscopy. This method allowed simultaneous measurement of the recovery dynamics of mCherry and GFP tags within FAs.

Initial experiments with A2058 cells confirmed intrinsic elevated Talin cleavage levels, as evidenced by a gap between the FRAP recovery curves of GFP and mCherry. This gap was significantly reduced upon treatment with the calpain inhibitor ALLN or transfection with a dual calpain site mutant of Talin (Talin DM). Repeat experiments in HFF cells, A375P cells, and A2058 cells consistently demonstrated larger gaps in transformed cell lines compared to HFF cells (Figure 3C, Left), further supporting the observation of increased Talin cleavage in transformed cells.

In conclusion, our findings suggest differential Talin cleavage levels between transformed and non-transformed cell lines, highlighting a potential role for Talin cleavage in cellular transformation processes.

### 3.4 Different Talin mutants affect transformed cell growth rate

**FIGURE 4:**
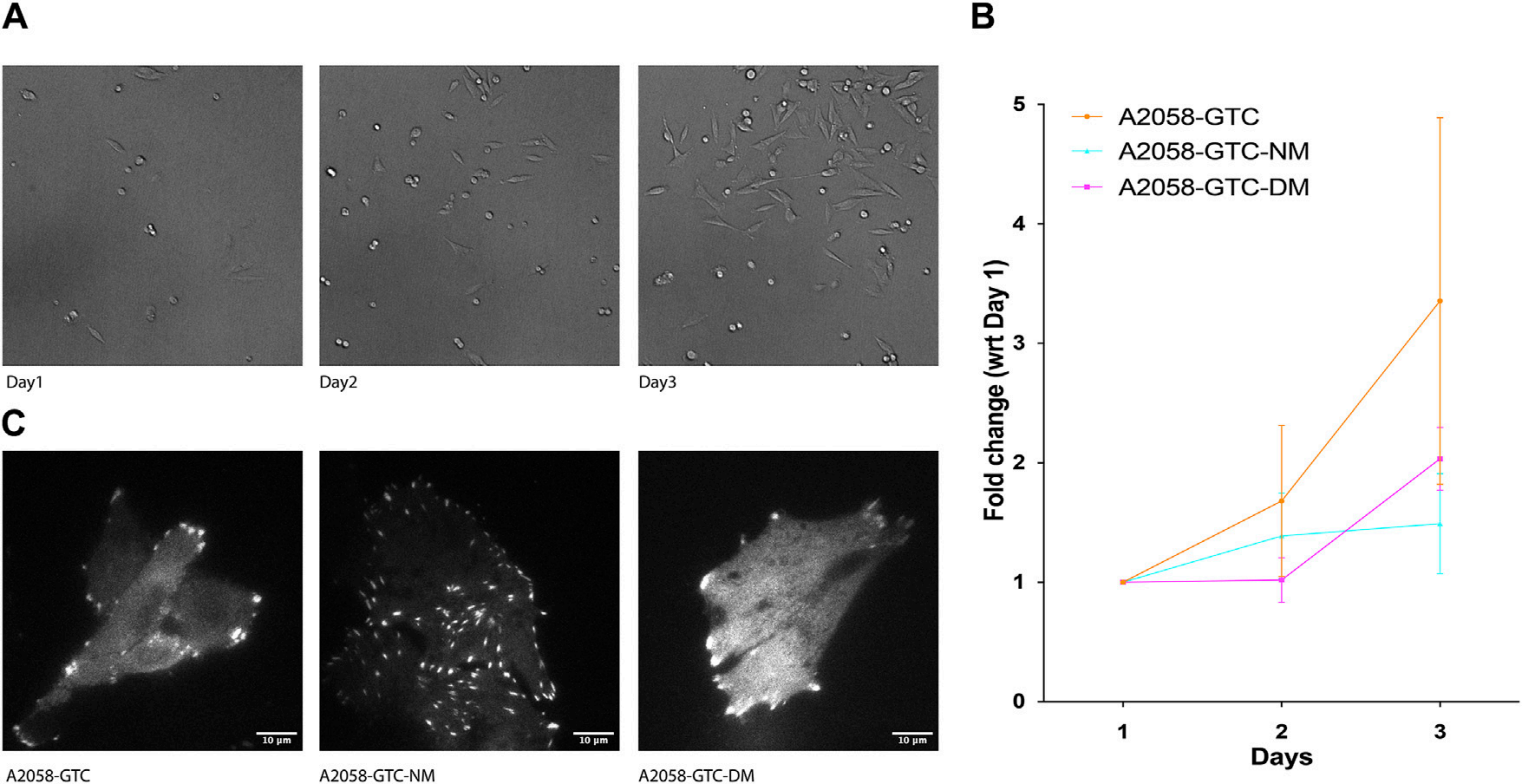
Expression of non-cleavable Talin-1 reduces A2058 cell proliferation.** (A)** Representative image showing A2058 cells transfected with different mutants seeded into 15.6 mm wells, which were used to count the number of cells for each condition daily over 3 days. **(B)** XY chart showing fold change in the number of cells normalized by the number of cells counted on the first day after seeding, plotted with standard deviation from at least 4 wells of seeded cells. **(C)** Representative TIRF microscopy image displaying A2058 cells transfected with GTC (Talin wild type), GTC-NM (N-terminus calpain site mutant), and GTC-DM (dual calpain site mutant). The fluorescence signal presented is a grayscale image of the GFP channel.

Previous studies have indicated that Talin cleavage can influence mechano-signal-induced cancer cell growth. To further explore this phenomenon, we conducted a growth assay using A2058 cells transfected with different Talin calpain mutants. Prior to transfection, the cells were silenced with siTLN1 to reduce endogenous Talin levels (Supplementary Figure S2). Subsequently, exogenous Talin constructs with three different mutations were introduced: GTC (wild-type Talin), GTC-NM (N-terminus calpain site mutant), and GTC-DM (dual calpain site mutant). Cells were seeded at 20% confluency on day 1, and the number of cells expressing positive fluorescence was counted on days 2 and 3. Remarkably, cells transfected with both calpain site mutants exhibited a significantly slower growth rate compared to those transfected with wild-type Talin, suggesting that reducing calpain cleavage levels attenuates transformed cell growth (Figure 4). This finding underscores the potential role of Talin cleavage in modulating the growth dynamics of transformed cells.

## 4 Discussion

In this current study we have employed dual fluorescent tagged Talin and it’s mutant to visualize FA morphology and dynamics in three melanoma cell lines (Meljuso, A375P and A2058) and a non-transformed cell line, human foreskin fibroblast (HFF). While the average number of FAs per cell did not significantly differ across the cell lines, notable differences were observed in the aspect ratio of the FAs and overall cell morphology. HFF cells exhibited significantly higher values for FA aspect ratio compared to the melanoma cell lines, indicating more elongated FAs in non-transformed than that in transformed cells. Less elongated FA can be the result of less mature FA, less contractile force and/or higher FA turnover rate. Total protein lysate analysis revealed significantly higher levels of endogenous Talin-1 in HFF cells compared to melanoma cells, suggesting a potential correlation between Talin-1 expression and FA characteristics.

Moreover, our study investigated the morphology of Talin calpain cleavage mutants in FA. We noticed that Talin calpain cleavage site mutants affect both the morphology of FA and cell. When the N-terminal calpain cleavage site is mutated, we observed an increase in FA assembly at the cell center, suggesting a role for this cleavage site in the disassembly of old FAs. Previous studies observed that the C-terminus cleavage site exhibiting lower calpain cleavage susceptibility. Conversely, our study shows that mutation of the C-terminal calpain cleavage site resulted in a higher detection of Talin in the cytosol compared to wildtype Talin which is highlighted by the blurred edge between FA and cytosol, indicating that this site is involved in localizing Talin to FAs. Importantly, although the C-terminus cleavage site mutation alters FA morphology by blurring the boundary, it does not abolish the localization of non-cleavable Talin within FAs. Furthermore, the shape of FAs and cell spreading area remain unchanged compared to wild-type Talin, indicating that the C-terminus cleavage site mutation indeed has minimal impact on FA rigidity sensing.

We next studied Talin cleavage levels in non-transformed (HFF) and transformed (Meljuso, A375P, A2058) cell lines using various analytical approaches. Western blot analysis and ratiometric fluorescence intensity measurements revealed higher Talin cleavage levels in transformed cell lines compared to HFF cells, indicating differential Talin cleavage levels between transformed and non-transformed cell lines.

The monoclonal antibody (97H6, Bio-Rad) for Talin1 can detect not only both isoforms of Talin, Talin1, Talin2, it can also detect cleaved Talin rod; and as a result can serve as a quick test to evaluate the Talin cleavage level. The intensity measurement shows that although all the three transformed cell lines have lower overall Talin expression level compared to HFF cells, they have slightly higher ratio of Talin rod indicating higher cleavage level. But the Western blot result has its clear limitation to detect Talin cleavage level in terms of detection precision as well as location specific detection. Being able to evaluate the Talin level with high precision and certainty is crucial to understanding the process.

Hence we introduced a second method, the ratiometric measurement of the fluorescence intensity. This method evaluates the relative protein level in a quantitative fashion, as long as the fluorescent emission is a linear process (i.e., more fluorescence protein density is proportional to photon emission at constant illumination). This relationship holds as long as the proteins are not that dense that the illumination light will noticeably be attenuated, a phenomenon that is very uncommon under physiological circumstances. Modern detectors (PMT, Avalanche diodes, EMCCD, sCMOS, etc.) are sensitive throughout the range of the visible spectrum and they respond very linearly (readout is linearly correlated to photons detected) unless the detector is saturated, an artifact easily avoided and very noticeable if it occurred, as the image goes into saturation. Using only ratios between different spectra channels, removes all first order dependencies on these parameters and leaves us with a robust quantification tool with a large dynamic range. Having said that, the absolute readout of the fluorescence intensity is still affected by instrument settings such as detector gain level, laser stability, filter efficiency, etc. TIRF based ratiometric imaging can also be affected by penetration depths and evenness of the evanescent field both of which can be affected by the laser incident angle, cleanliness of all optical elements along the light path, cover glass thickness variation, and of course local cell membrane flatness.

FA turnover is a highly dynamic process and sensitive to location. FA growth usually is most active at the leading edge of the cell and the disassembly is usually most active at the trailing edge. Fluorescence imaging is also a powerful tool to study dynamics of proteins in live cells. We have evaluated the use of Fluorescence Recovery After Photobleaching (FRAP) experiments to further investigate the Talin cleavage difference which is reflected in the protein turnover rate. Unlike intensity based rationmetric imaging, FRAP measures changes in a single channel with speed and extent of recovery being the readout, hence insensitive to factors that can affect fluorescence intensity.

In this experiment, we take the advantage of our TIRF dual camera FRAP system to FRAP the same FA in Green and Red channels in TIRF mode simultaneously. For GFP-Talin-mCherry, if the Talin is cleaved, the GFP tagged Talin head and mCherry tagged Talin rod should have different recovery half time (tau). Our control experiment shows that there is indeed a reduced difference in recovery half life of mCherry channel in the negative control groups of Talin cleavage (ALLN treated and Dual mutated cleavage site Talin) compared to that of the wild type Talin. This indicates that FRAP can be used as a powerful and sensitive method for detecting cleavage differences. We then applied the FRAP experiment to our dual tagged Talin in transformed and non-transformed cell lines. There is a bigger gap between the GFP and mCherry recovery curve in transformed cell lines than that of the non-transformed cell lines indicating that there is indeed an increased level of Talin cleavage in transformed cell lines. Each of the experiments were repeated more than 20 times on FAs that are located at the peripheral of cells in the same group. The individual variation of the recovery time within the same group is rather large. This is probably due to the fact that FAs at different cellular locations undergoes different levels of FA turnover which is indicated by calpain cleavage level. Cell type differences and force loading differences could result in variations in the FRAP recovery time of a particular FA. The following publication from Ballestrem’s group has demonstrated that different substrate rigidities can affect the FRAP recovery of most FA proteins (Stutchbury et al., 2017).

The ability to categorize FA based on their location within the cell, such as those on the leading or trailing edge, would be highly valuable for researchers studying cell migration and adhesion dynamics. Traditional methods relying solely on morphology are often insufficient due to the rapid turnover of lamellipodia. However, the fluorescence recovery after photobleaching (FRAP) experiment, which evaluates FA cleavage levels, offers a promising avenue for this categorization. The advantage of FRAP lies in its ability to provide quantitative data on the dynamics of FAs within a short timeframe, typically around 30 s in our experiment. This rapid assessment allows for the capture of real-time information about FA turnover and cleavage levels. Importantly, since FAs on the leading and trailing edges of cells are subjected to different mechanical forces and signaling cues, they may exhibit distinct cleavage patterns. By analyzing the FRAP profiles of FAs, researchers may be able to reliably categorize them based on their location within the cell.

This method holds great potential for future studies aiming to dissect the functional roles of FAs in cell migration and adhesion. By accurately characterizing FAs based on their cleavage levels, researchers can gain insights into the spatiotemporal regulation of FA dynamics and its impact on cellular behaviors such as migration and invasion. Moreover, this approach may facilitate the identification of specific molecular mechanisms underlying FA turnover and signaling in different cellular contexts. Overall, the integration of FRAP-based categorization of FAs represents a promising direction for advancing our understanding of cell adhesion dynamics and its implications in various physiological and pathological processes.

Lastly, we evaluated the effects of different Talin calpain mutants on FA morphology and cell growth dynamics in A2058 melanoma cells. Consistent with previous studies, cells transfected with calpain site mutants exhibited significantly slower growth rates compared to those transfected with wild-type Talin, suggesting a potential role for Talin cleavage in attenuating transformed cell growth. These findings highlight the intricate relationship between Talin cleavage, FA morphology, and transformed cell behavior, providing valuable insights into the mechanisms underlying cellular transformation processes.

In conclusion, our study sheds light on the intricate relationship between Talin cleavage, FA morphology, and transformed cell behavior. By elucidating the mechanisms underlying FA dynamics and cellular transformation, our findings contribute to a better understanding of the complex interplay between cellular architecture and signaling pathways in cancer progression. Further investigations into the role of Talin cleavage in modulating cellular behavior hold promise for the development of novel therapeutic strategies targeting cancer cell growth and metastasis.

## Data Availability

Raw data of FRAP experiments will be uploaded to BioImage Archive. DOI to raw data : https://doi.org/10.6019/S-BIAD1201.
