# Supplementary material for: Differential Talin cleavage in transformed and non-transformed cells and its consequences
Source: Front Cell Dev Biol. 2024 Jul 17;12:1430728. doi: 10.3389/fcell.2024.1430728 (PMC11289324; doi:10.3389/fcell.2024.1430728)
Supplement: Supplementary file 3 [file Presentation1.PPTX]

## Slide 1
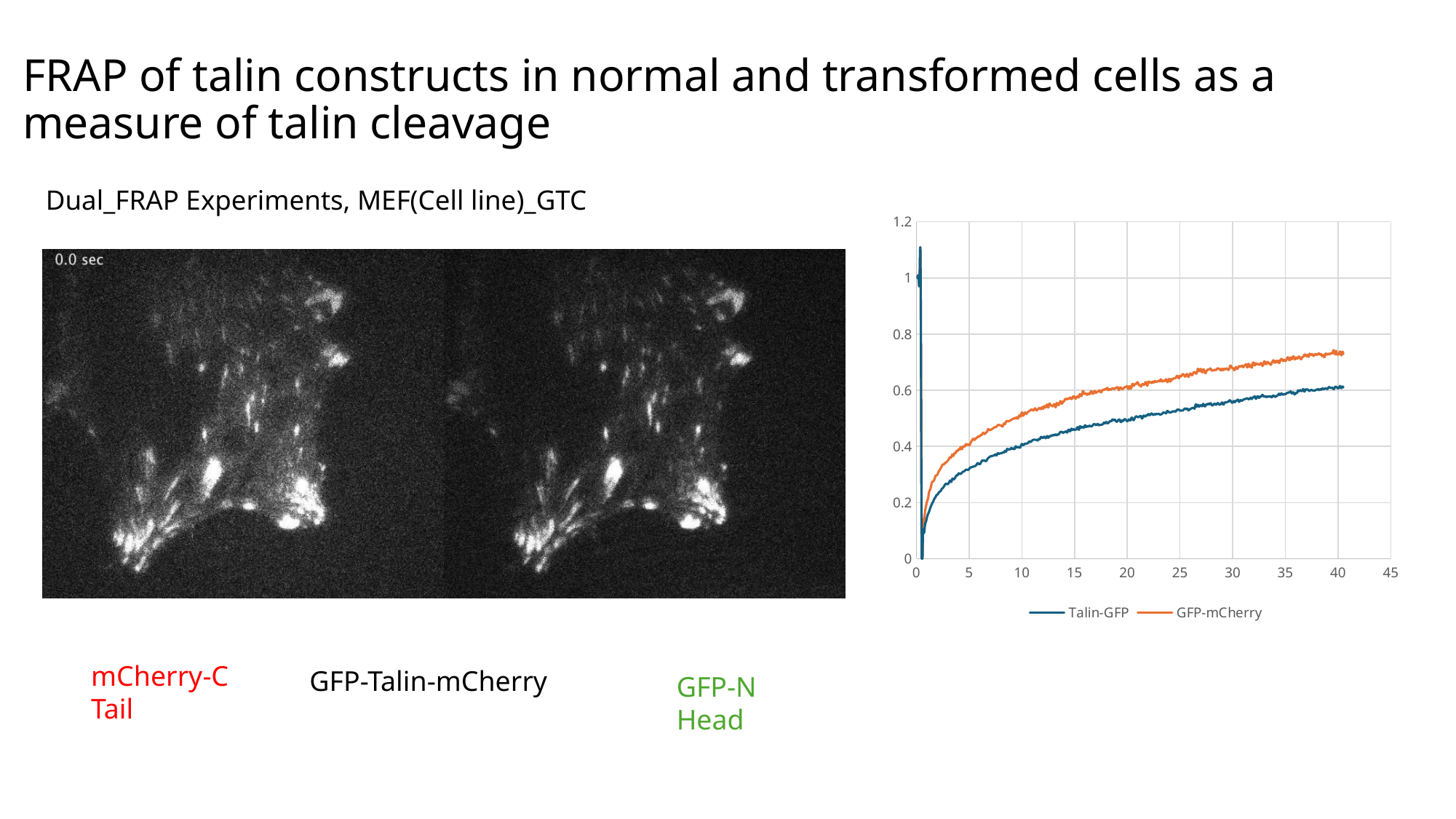

FRAP of talin constructs in normal and transformed cells as a measure of talin cleavage
# Dual_FRAP Experiments, MEF(Cell line)_GTC
### Chart
| Category | Talin-GFP | GFP-mCherry |
|---|---|---|mCherry-C
Tail
GFP-Talin-mCherry
GFP-N
Head
