# Supplementary material for: Differential Talin cleavage in transformed and non-transformed cells and its consequences
Source: Front Cell Dev Biol. 2024 Jul 17;12:1430728. doi: 10.3389/fcell.2024.1430728 (PMC11289324; doi:10.3389/fcell.2024.1430728)
Supplement: Supplementary file 5 [file Image1.pdf]

Supplementary

Supplementary Video 1

Representative Image of Dual FRAP Experiment of MDA231 Cells transfected by GFP-Talin-mCherry (100ms/frame). The MDA231 cells exhibit hyperactive lamellipodia and a highly diverse focal adhesion (FA) FRAP phenotype, which cannot be adequately summarized by simple averaging. Therefore, the data from these cells were not included in this study.

Supplementary Video 2

Representative Image of Dual FRAP Experiment of A375P Cells transfected by GFP-Talin N Terminal Mutation- mCherry (100ms/frame)

Supplementary Video 3

Representative Image of Dual FRAP Experiment of HFF Cells transfected by GFP-Talin-mCherry (100ms/frame)

Presentation 1

Overview of dual FRAP experiment on FA and data analysis

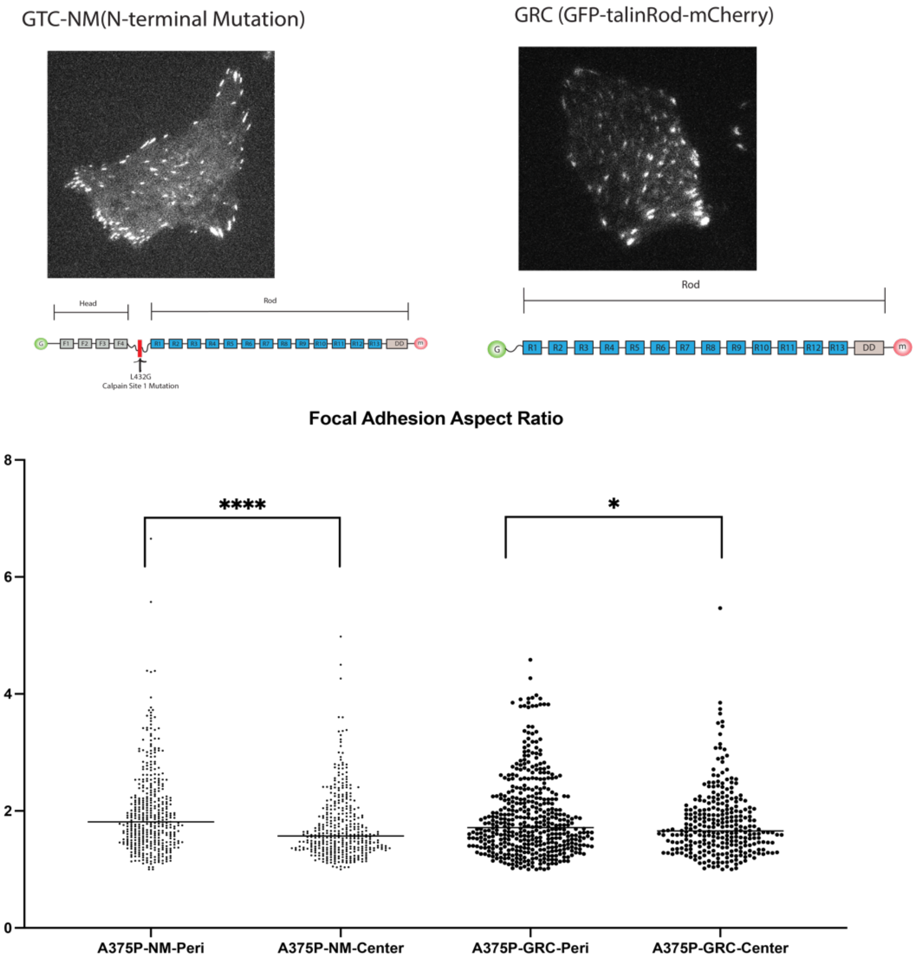

Figure S1. Differences in Focal Adhesion Morphology Between the Cell Periphery and Center. A375P cells transfected with GTC-NM (N-terminal mutation, L432G) and GRC (Talin rod) were analyzed. Focal adhesions located at the cell edge have a larger aspect ratio (AR) compared to those in the center. The difference in AR between peripheral and central focal adhesions is larger in GTC-NM (N-terminal mutation, L432G) compared to GRC (Talin rod). A larger aspect ratio indicates more elongated focal adhesions, which typically suggests they are under greater force.

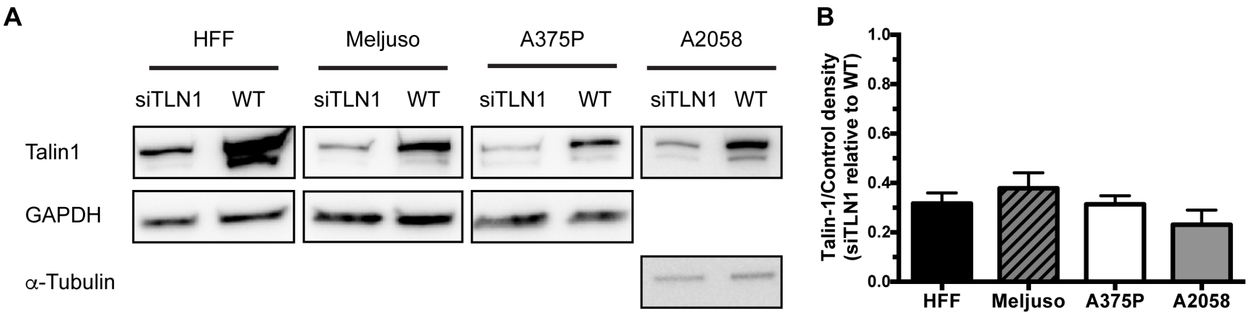

Figure S2. Genetic silencing of TLN1 in fibroblasts and melanoma cell lines. (A) Representative western blot images showing protein expression levels of Talin-1, GAPDH and α-Tubulin in total protein lysates taken from HFF, A375P, Meljuso and A2058 cells without (WT) and with TLN1 siRNA treatment (siTLN1). (B) Bar chart showing the ratio of Talin-1 expressed in cells treated with TLN1 siRNA to the expression in wild type cells normalized by loading control (GAPDH or α-Tubulin) plotted with mean and standard deviation from at least 2 blots.
